# Subject Based Registration for Individualized Analysis of Diffusion Tensor MRI

**DOI:** 10.1371/journal.pone.0142288

**Published:** 2015-11-18

**Authors:** Asif K. Suri, Roman Fleysher, Michael L. Lipton

**Affiliations:** 1 Department of Radiology, Albert Einstein College of Medicine, Bronx, New York, NY, United States of America; 2 Department of Psychiatry, Bronx Psychiatric Center, Bronx, New York, NY, United States of America; 3 Department of Radiology, Montefiore Medical Center, Bronx, New York, NY, United States of America; 4 Department of Psychiatry and Behavioral Sciences, Albert Einstein College of Medicine, Bronx, New York, NY, United States of America; 5 The Dominick P. Purpura Department of Neuroscience, Albert Einstein College of Medicine, Bronx, New York, NY, United States of America; University of Minnesota, UNITED STATES

## Abstract

Registration of subject and control brains to a common anatomical space or *template* is the basis for quantitatively delineating regions of abnormality in an individual brain. Normally, a brain atlas is chosen as the template. Limitations in the registration process result in persistent differences between individual subject brains and template, which can be a source of error in an analysis. We propose a new approach to the registration process where the *subject of interest* is the registration template. Through this change, we eliminate errors due to differences between a brain template and a subject’s brain. We applied this method to the analysis of FA values derived from DTI data of 20 individual mTBI patients as compared to 48 healthy controls. Subject-centered analysis resulted in identification of significantly fewer regions of abnormally low FA compared to two separate atlas-centered analyses, with subject-centered abnormalities essentially representing the common subset of abnormal low FA regions detected by the two atlas-centered methods. Whereas each atlas-centered approach demonstrated abnormalities in nearly every subject (19/20 and 20/20), the subject-centered approach demonstrated abnormalities in fewer than half the subjects (9/20). This reduction of diffusion abnormalities observed using the subject-centered approach is due to elimination of misregistration errors that occur when registering the subject of interest to a template. Evaluation of atlas-centered analyses demonstrated that 9.8% to 13.3% of subject GM and CSF was misregistered onto the WM of the brain atlas, resulting in the observation of additional low FA clusters compared to the subject-centered approach. Without careful evaluation, these misregistrations could be misinterpreted as pathology. An additional benefit of the subject-centered approach is that diffusion abnormalities can now be visualized directly in the subject’s anatomical space, rather than interpolating results from the brain atlas space, and can thereby enhance correlation with other components of an imaging protocol.

## Introduction

Conventional neuroimaging techniques, such as CT and MRI, readily demonstrate distinct lesions due to many neurological diseases. However, these techniques provide a limited window into brain pathology. Advanced MR imaging techniques, such as DTI, reveal additional pathology and offer important additional information that compliments standard imaging. It is increasingly apparent that even small changes in diffusion parameters can indicate clinically significant pathology. However, the inherent spatial variability of normal MR-derived diffusion measures limits the identification of significant alterations in these parameters by visual inspection. Thus, a quantitative approach to the evaluation of these data is essential. Quantitative methods for detecting regions of abnormal diffusion **in a single subject** require comparison of diffusion parameter images from the subject to the diffusion parameter images from a normative reference group. In order to ensure that analogous brain regions are compared, one of two basic strategies is followed: a region-of-interest (ROI) based approach or a voxel-based approach [1–4].

In ROI based approaches, brains of the subject and controls are segmented into anatomical structures and the average values of the metric of interest within each ROI are compared. ROIs are commonly delineated based on anatomical landmarks or by registering subjects’ brains to a parcellated template. Use of larger ROIs has been proposed as a means to mitigate registration errors in ROI based analyses [5]. However, modifying the size of the ROI does not in fact alter the registration process or its accuracy. For a given registration algorithm, inaccuracy may be less conspicuous when averaged over a larger area, but will be present to the same degree whether the image volume is analyzed at the level of one or many voxels. A more robust implementation of this approach deforms an atlas-based template to each subject, thereby creating an individualized set of anatomical ROI for each individual [5]. However, the implicit assumption of any ROI based approach is that boundaries of abnormalities coincide with boundaries of delineated ROIs and affect most of the tissue within the ROI in the same way. In many diseases, including mTBI, foci of pathology cannot be expected to respect and be delimited by canonical anatomical boundaries. Thus, the implicit assumption of the ROI based analysis cannot be expected to hold and the use of ROI-based methods to detect abnormalities is associated with penalties in sensitivity due in large part to partial volume effects and cancelations by spatial averaging. To improve the detection of abnormalities in a single subject for a given ROI, enlarging the normative cohort may offset the loss of sensitivity.

In voxel-based approaches, subject and control images *must be* registered to a template, placing each brain into a common reference frame, after which a voxel-by-voxel comparison over the entire brain is performed. Since no region-specific assumption is made, this method is inherently more sensitive to abnormalities in a single subject as compared to the ROI-based approach. Typically, a brain atlas, such as those available from the Montreal Neurological Institute (MNI) [6,7] or Johns Hopkins University (JHU) [8–10], is chosen as the template for this “atlas-based registration” (aBR) process. Such *atlas-centered analysis* of DTI data is typically displayed as a color overlay on T1-weighted images (T1WI) of the brain atlas template.

**Fig 1:**
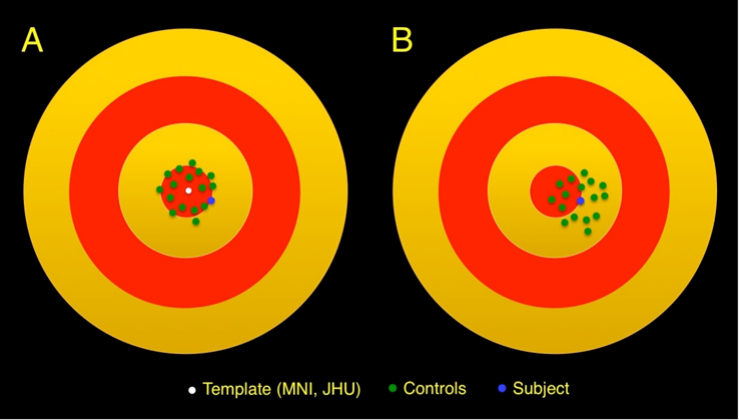
Schematic diagram representing the conformational space of the brain (concentric red and yellow circles). (A) When the target of the registration process is a brain atlas template, registration of the controls (green dots) and subject (blue dot) result in a distribution of brain conformations around the brain atlas template (white dot). (B) If the target of the registration process is the subject, registration of the controls results in a distribution of brain conformations around the patient.

Registration processes are limited in the degree to which the anatomy of one brain can be mapped on to another brain [11]. Although appropriately registered brains closely resemble a chosen template, they are not anatomically identical and therefore, constitute a distribution in conformational space around the template (Fig 1A). Consequently, voxels from individual subjects’ diffusion parameter images closely but not exactly map onto corresponding voxels of the template. Therefore, some regions of the subject’s brain may be classified as abnormal not because there is abnormality but because disparate brain regions are compared between subject and controls. Conversely, an abnormality may be missed if a truly abnormal value happens to fall within the normal range at the control brain region to which it is improperly compared.

Numerous registration algorithms have been developed in an effort to improve the accuracy in registering a subject brain to a given template, some faring better than others [11]. In addition, a method of asymmetric image-template registration [12] and a study of the effects of registration regularization and atlas sharpness on segmentation accuracy [13] have been performed to improve the registration process. Although improvements in the registration process can be made, no algorithm is likely to perfectly register one brain onto another; some level of limitation will persist with respect to accuracy of the registration result. Because registration quality is sensitive to the similarity of subject brains to the template, one method to increase accuracy is to use a study-specific “central” template—a template requiring least average deformation of the brains in the study—with the idea that smaller deformations are accompanied by smaller registration errors [14]. Since mapping of voxels from an individual brain to a template is not precise, voxel-based approaches suffer sensitivity loss due to broadening of the distribution of any quantitative metric by inclusion of values from other locations, manifesting as additional noise in the metric at each voxel [11]. While more accurate deformation algorithms and use of a central template lead to smaller broadening, further noise reduction by increasing the number of individual brains and thereby further averaging inherent registration inaccuracies, as in the ROI-based analysis (above), is always available. This approach works well in both ROI-based and voxel-based group-to-group comparisons because averaging takes place within each group.

Limitations of the registration process in the voxel-based analysis manifest very differently when comparing a single subject to a group of controls: the averaging effect described above is operative for the controls, but not for the subject. In this situation, imperfect registration of the subject to the template leads to a bias: a diffusion metric derived from the subject is compared to values from controls at a different anatomical location, exclusively due to misregistration of the subject to the template (Fig 1A). Moreover, because the quality of registration is sensitive to how similar a subject is to the template, the bias depends on choice of template. Increasing the number of controls cannot reduce this bias. Although data from the subject and controls appear to be handled identically, as the registration analysis steps are the same, the subject’s data is in fact treated differently, since averaging of registration errors occurs only for controls. To remove this bias, the subject’s image must be explicitly treated differently in order to put it on equal footing with those of the control group.

In this study, we exploit the sensitivity of this bias to the choice of the template and propose a modification of the registration scheme for voxel-wise comparison of a single subject to a group of controls: choosing the individual *subject of interest* as the template (Fig 1B). Such subject-based registration (sBR), which we term *SUbject-REgistered Quantification* of DTI (SURE-Quant DTI), thus occurs in the subject’s anatomical space. Due to limitations of the registration process, errors of registration of each control to the subject (serving now as the template) are still present. However, these errors average across controls, as is in the conventional atlas-centered approach. Most importantly, these errors will not affect the subject. We hereby eliminate bias inherent when atlas-based registration (aBR) is used to compare a single subject to a group of controls. We demonstrate dependence of this bias on choice of template and the impact of the SURE-Quant approach.

## Material and Methods

After Albert Einstein College of Medicine Institutional Review Board approval and written informed consent, a cohort of 20 acute mTBI patients were prospectively recruited from an urban Emergency Department (ED), distinct from clinical care [3,15]. A total of 48 healthy individuals were recruited from the local community through advertisements as part of the same study [3,15]. Inclusion criteria for patients included: age 18–70 years, ED diagnosis of concussion within 2 weeks, Glasgow Coma Scale = 13–15, loss of consciousness<20 minutes, posttraumatic amnesia<24 hours, no focal neurologic deficits and proficiency in English or Spanish. Exclusion criteria for patients and controls included: prior head injury, hospitalization due to the injury, neurodevelopmental or neurological disorder, major psychiatric disorder, illicit drug use within 30 days, or skull fracture or abnormal CT [3,15]. Prior head injury was determined by questioning subjects if they ever suffered a head injury “for which they sought or were advised to seek medical attention.” Subjects were also excluded if ED records indicated prior concussion or TBI.

All patients and controls underwent 3 Tesla diffusion tensor MRI as well as acquisition of T1WI and T2-weighted images (T2WI) on a Philips Achieva TX scanner using an 8-channel head coil. T1WI was performed using 3D MPRAGE (TR/TE, 9.9/4.6 ms; field of view, 240 mm; matrix, 240x240; section thickness, 1 mm). T2WI was performed using axial 2D TSE (TR/TE, 4000/100 ms; field of view, 240 mm; matrix, 384x512; section thickness, 4.5 mm). DTI was performed using single shot EPI (TE, 51ms; flip angle, 90 degrees; section thickness, 2mm; matrix = 128x128; FOV, 256x256; diffusion directions, 32; b value, 800 sec/mm^2^).

Removal of non-brain voxels, eddy current correction, tensor fitting and rigid body registration of the diffusion data to the individual’s T1WI were performed using the FMRIB-FSL software package [16–18]. EPI distortion correction of each diffusion tensor MRI dataset was performed with the Automated Registration Toolbox package using a non-linear procedure [19,20]. The distortion-corrected EPI volume thus produced was then registered to the patient’s T1W volume, using a rigid body transformation, as a separate step. For the aBR approach, T1W was registered to the template brain (MNI, JHU) using the 3D-Warper module from the Automated Registration Toolbox [19,20].

Three sets of identical analyses comparing each subject to the entire control group, with each employing a different template, were performed: (1) all individuals were registered to the MNI template (aBR-MNI), (2) all individuals were registered to the JHU template (aBR-JHU) and (3) for each subject’s analysis, all controls were registered to the subject’s brain (sBR). It should be emphasized that in this latter approach, the template was different for each subject’s analysis, with that template being the subject’s brain.

Each subject’s registered T1WI was segmented using the fast automated segmentation tool (FAST) within the FMRIB-FSL package [16–18], yielding binary masks of WM, GM and CSF. WM masks of the template brain for each analysis (MNI, JHU or patient) were used to restrict the subsequent voxelwise comparison of FA images to WM voxels only. A voxelwise *t* test was performed comparing each patient’s FA to that of the 48 control subjects. Clusters of abnormally low FA voxels were defined by applying a voxelwise threshold of p < 0.005. Clusters that reached a minimum size of at least 100 contiguous voxels were considered significant in this study (see S1 Appendix). These criteria have been previously reported [21]. It should be emphasized that the registration procedure and voxelwise analysis were applied in the same way for each of the three analyses. The analyses differed only in the template chosen for the registration process.

In order to assess whether the numbers of abnormally low FA clusters detected using the sBR approach were significantly different from those detected using either of the aBR methods and to compare the aBR approaches to each other, a paired Wilcoxon signed-rank W-test was performed.

In addition, to demonstrate that the observed differences are not specific to mTBI and to establish base rates, we selected two independent groups of 20 healthy subjects. The first set of 20 subjects was labeled the “reference group” while the second the “test group”. We then repeated the sBR and aBR-JHU analyses for each mTBI and each test subject against the reference group.

The GM and CSF masks derived from each subject’s registered T1WI were combined to produce a single mask delineating all “non-white matter” brain voxels. This mask was used to identify regions where non-white matter of a registered subject brain was misregistered and overlapped with white matter voxels of the template. We term these GM/CSF misregistrations.

## Results

### Number of Low FA Clusters

Table 1 shows the number of abnormally low FA clusters detected in each of the 20 mTBI patients, using each of the three registration methods while Table 2 shows the number of abnormally low FA voxels within clusters. Locations of these clusters tend to be in regions typical of mTBI injury [22,23]. Both aBR approaches detected significantly more abnormally low FA clusters (aBR-MNI = 90; aBR-JHU = 101) than the sBR approach (sBR = 24), thus detecting an average of 76.8% fewer abnormally low FA clusters compared to the two aBR methods. The results of the Wilcoxon signed-rank test identified significant differences between the number of abnormally low FA clusters in each of the 20 subjects using the sBR method to those identified in each using the aBR approach (both sBR vs aBR-JHU and sBR vs aBR-MNI had W = 171 (N = 18) one-tailed p-value < 0.0001). In contrast, the number of abnormally low FA clusters identified with the two aBR methods did not differ (W = 34 (N = 16) one-tailed p-value = 0.1922). Whereas the aBR approaches detected clusters of abnormally low FA voxels in nearly all subjects (19/20 and 20/20 for MNI and JHU spaces, respectively), the sBR approach detected the abnormality in less than half the patients (9/20). On average, the aBR approaches detected 3.3 and 3.9 more clusters of abnormally low FA voxels per subject (MNI and JHU based templates, respectively) compared to the sBR approach. For the 9 subjects in whom abnormally low FA clusters were detected using the sBR approach, 383.3% to 405.5% more FA voxels were identified as having abnormally low FA when the aBR approaches were used (aBR-MNI and aBR-JHU approaches, respectively).

**Table 1:** Abnormally Low FA Clusters.

Patient	Low FA Clusters (sBR)	Low FA Clusters (aBR-MNI)	Low FA Clusters (aBR-JHU)	Overlap Low FA Clusters (sBR vs aBR-MNI)	Overlap Low FA Clusters (sBR vs aBR-JHU)	Overlap Low FA Clusters (aBR-MNI vs aBR-JHU)*
TBI1	0	3	2	0	0	0
TBI2	0	4	3	0	0	0
TBI3	2	4	2	1	1	0
TBI4	1	4	7	1	1	1
TBI5	4	8	8	4	4	0
TBI6	0	6	5	0	0	0
TBI7	0	7	4	0	0	0
TBI8	2	6	4	2	2	0
TBI9	0	3	6	0	0	0
TBI10	1	10	7	0	1	0
TBI11	0	5	10	0	0	1
TBI12	0	1	2	0	0	0
TBI13	0	0	2	0	0	0
TBI14	0	2	4	0	0	0
TBI15	6	9	8	6	6	0
TBI16	1	5	5	0	1	0
TBI17	0	1	7	0	0	0
TBI18	0	1	4	0	0	0
TBI19	3	7	7	3	3	1
TBI20	4	4	4	3	3	0
**SUM**	**24**	**90**	**101**	**20**	**22**	**3**

* Excluded were aBR clusters that overlapped with either sBR clusters or GM/CSF misregistrations

**Table 2:** Number of Abnormally Low FA Voxels within Clusters.

Patient	Low FA Voxels (sBR)	Low FA Voxels ^+^ (aBR-MNI)	Low FA Voxels ^+^ (aBR-JHU)	Voxel Increase^*^ (aBR-MNI)	Voxel Increase^*^ (aBR-JHU)
TBI1	0	415	247	**415**	**247**
TBI2	0	472	667	**472**	**667**
TBI3	210	645	270	**435**	**60**
TBI4	193	802	1089	**609**	**896**
TBI5	3312	6190	4695	**2878**	**1383**
TBI6	0	2065	710	**2065**	**710**
TBI7	0	4270	2477	**4270**	**2477**
TBI8	778	1606	1414	**828**	**636**
TBI9	0	865	1120	**865**	**1120**
TBI10	127	1473	1711	**1346**	**1584**
TBI11	0	1339	2836	**1339**	**2836**
TBI12	0	215	328	**215**	**328**
TBI13	0	0	345	**0**	**345**
TBI14	0	400	926	**400**	**926**
TBI15	1433	2361	2015	**928**	**582**
TBI16	146	861	1114	**715**	**968**
TBI17	0	523	1943	**523**	**1943**
TBI18	0	111	1423	**111**	**1423**
TBI19	404	1297	1101	**893**	**697**
TBI20	938	928	1020	**-10**	**82**
**AVG**				**965**	**996**

^+^ Volume adjusted to Patient Volume

^*^ Number of Additional Low FA Voxels Compared to sBR

When comparing against the 20-subject reference group (above), only 2 test subjects and only 1 mTBI patient showed no abnormalities in the aBR-JHU analysis, whereas these numbers become 10 and 10 in the sBR analysis. This difference is significant (test sBR vs test aBR: W = 140, N = 17, one-tailed p < 0.0005 and mTBI sBR vs aBR: W = 194, N = 20, one-tailed p < 0.0002). These results for mTBI subjects are similar to those obtained using the full set of controls (n = 48). We note that the observed rate of abnormalities in the test group is very similar to that of the mTBI subjects using either approach. This is because the mTBI patient sample we address does not lend itself to discriminating base rate in normal subjects from our patient sample. The large majority of these mildly injured patients, who we imaged during the acute phase, recover at 1-year follow-up. We therefore do not expect the rate of abnormalities in patients to be excessive.

**Fig 2:**
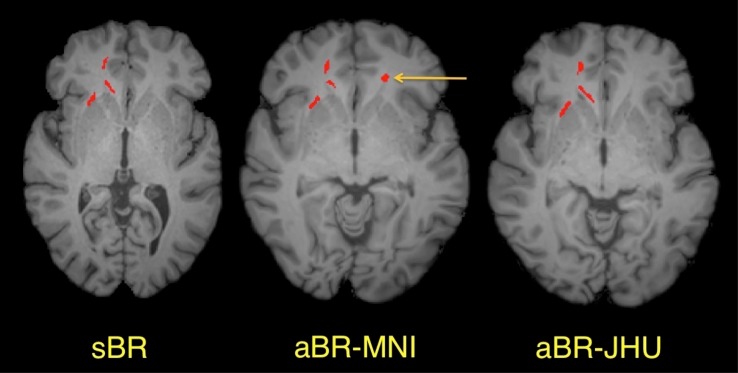
Abnormally low FA clusters detected in a single mTBI patient using three different registration approaches. Abnormally low FA clusters are shown in red overlaid on axial T1-weighted images: the patient’s anatomy (sBR), the MNI atlas (aBR-MNI) and the JHU atlas (aBR-JHU), respectively (left to right). Most abnormally low FA clusters observed using sBR were also present using the aBR methods, as demonstrated by the 3 clusters seen in the right frontal lobe. However, additional clusters (e.g., yellow arrow) detected using a particular aBR template generally will not overlap with the alternate aBR template or sBR.

Most clusters of abnormally low FA detected using the sBR approach were also detected using the aBR approaches (Fig 2, Table 1). Comparing the 24 clusters of abnormally low FA detected in 9 patients using the sBR approach, 20/24 (83.3%) were detected using the aBR approach with the MNI template, while 22/24 (91.7%) were detected using the aBR approach with the JHU template. In contrast, we observed very little overlap between those clusters detected using the two aBR approaches (excluding those clusters identified in the sBR approach or misregistered to non-WM regions). Specifically, when comparing the 70 clusters detected using the aBR-MNI approach to the 79 clusters detected using the aBR-JHU approach, only 3 clusters showed overlap.

**Fig 3:**
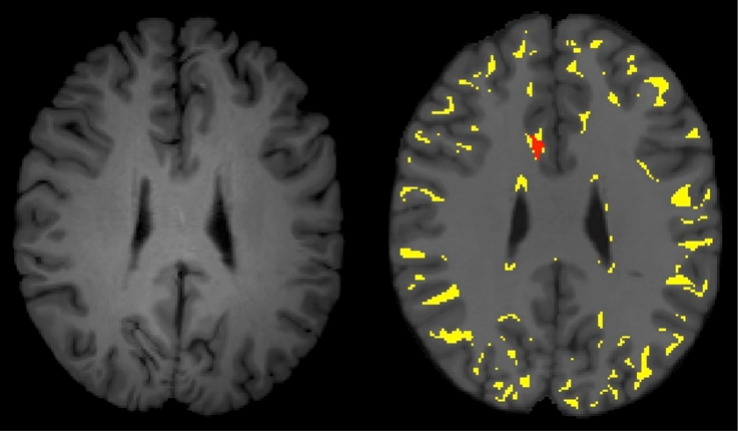
Misregistration of GM/CSF voxels onto WM of an atlas based template. On the left is an axial T1-weighted image of an mTBI patient transformed to match the MNI template. On the right is a corresponding T1-weighted axial image from the MNI template. The yellow overlay demonstrated the GM and CSF portions of the registered mTBI brain that have been misregistered to the WM of the MNI template. The red overlay is an abnormally low FA cluster that clearly incorporates misregistered voxels.

We quantified the extent of misregistration of GM or CSF as WM in each patient (Table 3). Using the aBR approach, 9.8% to 13.3% of GM/CSF voxels were misregistered to WM. Although many of these misregistered voxels do not result in FA abnormalities, 39.9% to 51.2% of voxels detected within abnormally low FA clusters are a result of such misregistrations (Fig 3 and Table 4). It is important to point out that the subject’s image cannot be misregistered when using the sBR approach, because the subject *is* the template and does not undergo registration. As a point of clarification, we refer to misregistration as displacement of tissue beyond the borders of a tissue interface, not partial volume effects. Partial volume averaging exists due to the finite size of the voxel. These effects cannot be eliminated by the sBR or any other analysis approach.

**Table 3:** Misregistration of GM/CSF onto WM of Template.

Patient	Number of WM Voxels (Patient)	Number of Misregistered GM/CSF Voxels on WM of Template (aBR-MNI)	Number of Misregistered GM/CSF Voxels on WM of Template (aBR-JHU)	Percent Overlap Misregistered GM/CSF Voxels and WM Template (aBR-MNI)	Percent Overlap Misregistered GM/CSF Voxels and WM Template (aBR-JHU)
TBI1	527752	75859	53688	12.5	9.5
TBI2	625444	80502	56677	13.2	10.0
TBI3	638950	79791	48797	13.1	8.6
TBI4	515464	80629	57413	13.3	10.1
TBI5	519619	83016	53590	13.7	9.4
TBI6	591886	81188	51184	13.4	9.0
TBI7	688909	82261	55971	13.5	9.9
TBI8	474424	82225	51525	13.5	9.1
TBI9	679664	79110	58091	13.0	10.2
TBI10	619657	79616	57949	13.1	10.2
TBI11	642122	72919	55168	12.0	9.7
TBI12	566821	76050	55562	12.5	9.8
TBI13	617823	75079	49328	12.4	8.7
TBI14	649398	74964	56431	12.3	9.9
TBI15	613713	89060	63087	14.7	11.1
TBI16	633477	73954	53026	12.2	9.3
TBI17	645457	93796	66432	15.4	11.7
TBI18	551298	80069	52994	13.2	9.3
TBI19	579439	94136	69478	15.5	12.2
TBI20	594236	79985	47348	13.2	8.3
**AVG**				**13.3**	**9.8**

Note: Number of WM voxels (MNI atlas) = 607633

Number of WM voxels (JHU atlas) = 567402

**Table 4:** Overlap of Voxels from Abnormally Low FA Clusters and GM/CSF Misregistered Voxels.

Patient	Percent Overlap (sBR)	Percent Overlap (aBR-MNI)	Percent Overlap (aBR-JHU)
TBI1	0	77.4	15.0
TBI2	0	86.9	77.5
TBI3	0	36.5	50.4
TBI4	0	23.8	40.6
TBI5	0	4.9	7.4
TBI6	0	20.9	65.5
TBI7	0	40.4	84.3
TBI8	0	14.0	15.7
TBI9	0	60.7	37.2
TBI10	0	47.4	28.6
TBI11	0	45.5	48.5
TBI12	0	4.8	100.0
TBI13	0	N/A^*^	93.1
TBI14	0	19.5	86.5
TBI15	0	5.9	16.5
TBI16	0	29.5	50.2
TBI17	0	90.9	72.8
TBI18	0	98.4	75.4
TBI19	0	37.4	43.6
TBI20	0	13.8	15.6
**AVG**	**0**	**39.9** ^*^	**51.2**

* No Abnormally Low FA Clusters in this aBR-MNI registration thus ratio is undefined. Average excludes this patient.

## Discussion

We demonstrate, for a voxelwise analysis comparing an individual subject to a group of controls, that simply defining the template as the subject themself results in a significant decrease in the number of low FA clusters detected. The additional clusters of abnormally low FA identified in analyses based on the aBR approach are primarily due to misregistration of the subject’s GM/CSF as WM during the registration process. This explains the lack of concordance of such clusters between the aBR-MNI and aBR-JHU approaches. These effects are solely due to the choice of registration target (brain atlas as template vs. subject as template) because all other aspects of the analysis remained identical; image data, image processing and statistical analyses were not changed across the different registration approaches (aBR vs. sBR). Therefore, these factors have no bearing on the effects we observe.

### Misregistration as the Source of Error

Using the aBR method, the WM mask of the template (MNI or JHU) defines those voxels to be included in the analysis, whereas in the sBR method, the WM mask of the subject’s brain (the template for sBR) defines those voxels to be included in the analysis. With the aBR approach, misregistration of the subject can lead to erroneous classification of gray matter or CSF containing voxels as white matter. As the set of controls are employed collectively to identify abnormal FA values in the subject, misregistration of an individual control will not significantly affect the determination of abnormally low FA voxels in a subject; these registration errors will be distributed across controls and have no significant net effect. However, misregistration of the voxels of the subject’s brain, to which the ensemble of controls are compared in the one vs. many analysis, can have a significant effect on the determination of abnormally low FA voxels. This error is eliminated using the sBR approach, where the subject’s anatomy is not transformed, but rather serves as the template for the analysis.

### Individual-to-Group vs. Group-to-Group Comparison: Reduction of Bias

In order for conclusions to be reliable when testing a hypothesis, the test must be constructed in a *fair* manner. When testing the role of a factor, the test is considered *fair* if that factor is changed while other aspects of the test remain the same; otherwise, the test may be biased. That is, significant findings cannot be attributed exclusively to the factor of interest. In a group-to-group comparison designed to characterize manifestations of a disease, care must be taken to collect and analyze data from both subjects and controls in the same manner, so that the factor under investigation is the sole variable. In this case, the aBR approach is the method of choice. If, however, the test is intended to detect evidence of disease in an individual, and not to provide the initial characterization of disease-related imaging changes, the principle of treating both subject and control groups in an identical manner would be inappropriate as the sample of interest is no longer a group, but a single individual, while the group of controls remains large. This unavoidable imbalance of group size threatens the fairness of the test and can lead to bias. And in fact, as we demonstrated, it actually does. The averaging of registration errors, as in a group-to-group comparison, cannot be achieved for the subject of interest in an individual-to-group comparison. However, registration of each control to the subject (the sBR method) does average registration errors across the group of controls and preempts these errors entirely in the subject of interest. Thus, sBR assures fairness of the comparison of single individual to a control group.

Misregistration of control images increases variance in the diffusion metrics with both aBR and sBR approaches. This issue can only be addressed by improving registration algorithms themselves. Notwithstanding this inherent limitation of all approaches, the sBR approach does eliminate bias (not variance) inherent in the aBR approach.

### Sources of Subject-to-Template Misregistration

Misregistration is the assignment of an anatomical location in the brain volume of a subject to a different anatomical location in the template brain. This is clearly evident when GM/CSF is misclassified as WM during the registration process. The comparison of GM/CSF (which inherently has lower FA than WM) to WM may yield erroneous inferences of abnormally low FA in the subject. Using the aBR approach, we found significant misregistrations of GM/CSF voxels onto the WM of the brain atlas template, with as much as half of these misclassified GM/CSF voxels contributing to observed clusters of abnormally low FA. It should be noted that WM could also be misregistered, either to GM/CSF or to other WM locations. Misregistration of WM to WM is more difficult to visualize as white matter generally has relatively homogeneous signal intensity on typical structural images. Thus, white matter voxels misregistered to adjacent white matter voxels appear similar to the native voxels at the location to which they are misregistered. Although the T1-weighted signal of these adjacent voxels might be similar, misregistration still can occur and therefore diffusion parameters at these adjacent locations can differ.

**Fig 4:**
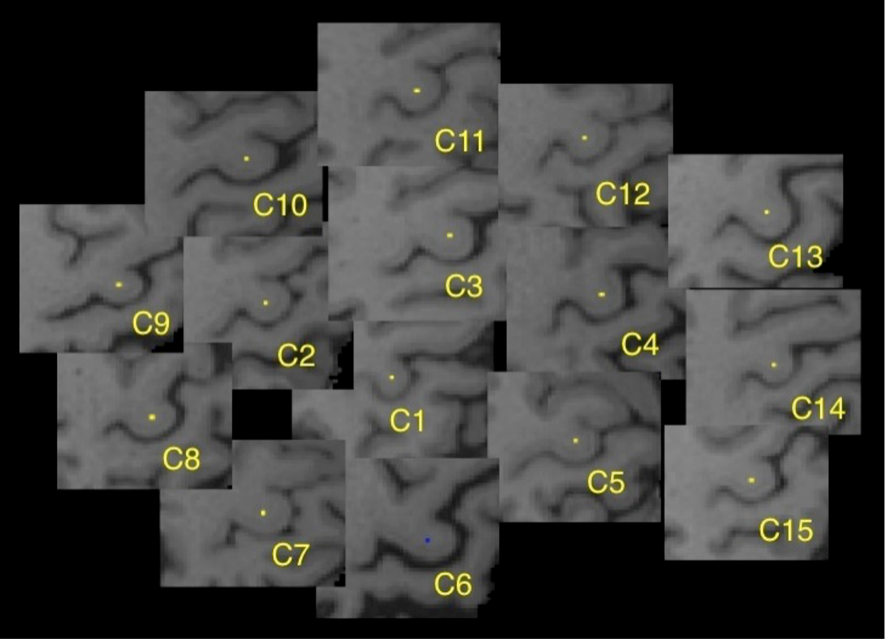
Tracking individual voxels through the registration process. Figure demonstrates a single labeled voxel within the *hand-knob* region of the left precentral gyrus in each of fifteen control subjects prior to transformation to the template.

**Fig 5:**
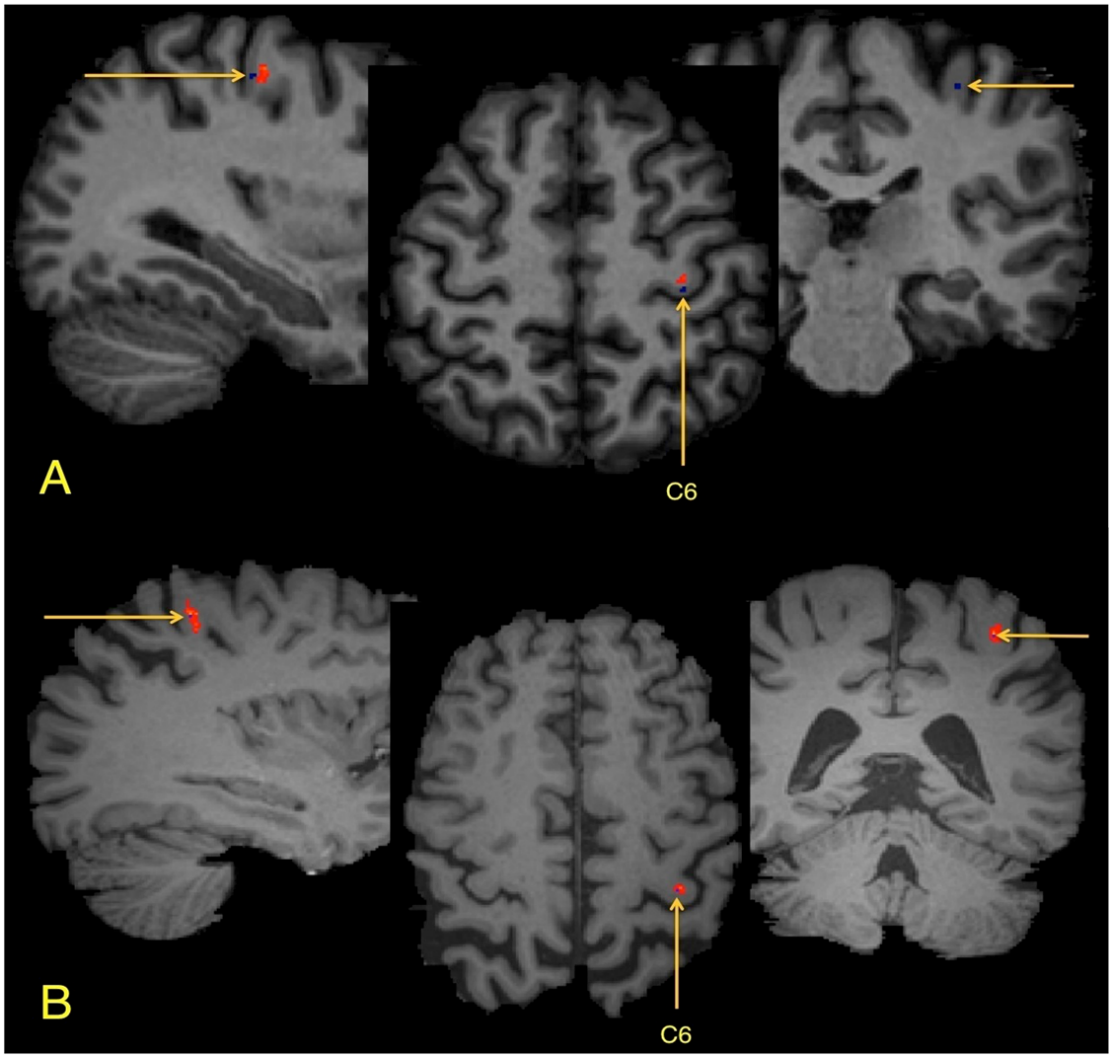
Demonstration of the registration result of aBR and sBR approaches. (A) Registration using the aBR-JHU method: all voxels are located within the hand-knob region, one voxel (blue), from control 6 (C6), has been displaced into GM. (B) Registration using the sBR approach, using C6 as the template, demonstrates the resultant location of these same 15 voxels. Using the sBR approach, the labeled voxel of C6 remains centered in the *hand-knob* gyrus with the labeled voxels of the other subjects, while still present in the gyrus, are distributed around the C6 voxel.

To illustrate the misregistration process, we can follow a single voxel as it is transformed to the template anatomy. Using 15 control subjects from this study, a single white matter voxel was identified and labeled, within the center of the “hand-knob”, a prominent anatomic feature of the precentral gyrus [24], in the left cerebral hemisphere of each control (Fig 4). Each of these control brains was then registered to the JHU template, as would occur in the aBR approach. The transformed labeled “hand-knob” voxel was then displayed as a color overlay on the JHU template brain image (Fig 5A). Examination of the registered voxels from each of the controls demonstrates that they were not transformed to the same central location within the “hand-knob”, but were distributed within the gyrus. This demonstrates the potential for error in the registration process. The transformed voxel of one control (control 6) was actually misregistered to the cortical gray matter at the surface of the “hand-knob” of the template brain. In order to simulate the sBR approach, we repeated this registration process, assigning as the template, the brain volume of the control whose brain was misclassified when registered to the JHU template (Control 6, above) (Fig 5B). The previously marked voxel remained centered within the “hand-knob” white matter because, serving as the template for the registration, this individual brain volume did not undergo any transformation. The labeled voxels of each of the remaining control subjects were distributed in proximity to the template voxel, again reflecting the inherent imperfection of the registration process.

Errors in accuracy using the aBR approach arise due to the misregistration of subject anatomy onto a brain atlas template. Such errors can lead to erroneous detection of abnormal FA clusters, which are the result of errors in the registration process, not of pathology. It is important to note that such erroneously detected clusters, are not present in the sBR results, but can be identified in the aBR results at locations where the subject’s white matter overlaps GM or CSF in the brain atlas template. Without careful scrutiny and sufficient interpretation experience, erroneous conclusions regarding the nature or even the presence of pathology can occur, when using the aBR methodology. Elimination of these erroneously detected clusters using the sBR approach can provide for more definitive determinations, which are less dependent on the expertise of those interpreting the results.

### Advantages of sBR

**Fig 6:**
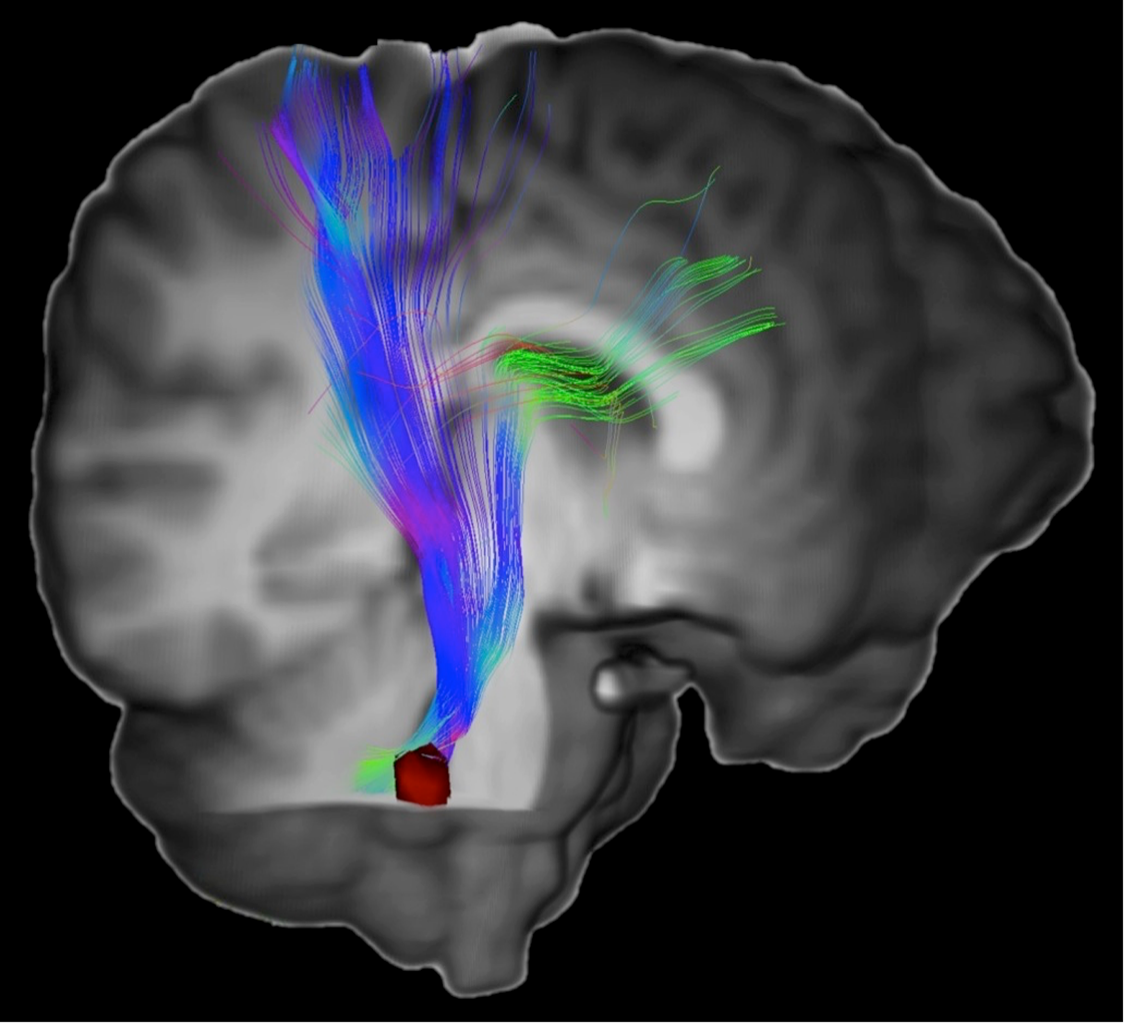
Diffusion tensor tractography in an mTBI patient based on sBR derived clusters. Overlying the 3D rendered T1WI a tractogram generated from the patient’s diffusion tensor imaging is shown in color. Tractography was performed using the Medinria software package (v1.8.0) and a single seed ROI within the right middle cerebellar peduncle (red). The seed ROI is a cluster of abnormally low FA generated from voxelwise analysis using the sBR approach. An additional ROI in the right cerebral peduncle was used as a waypoint.

The increase in accuracy obtained through use of the sBR approach is an important advantage of this method. Another important advantage is that the quantitative results may be viewed directly in the anatomical space of the subject. When using the aBR approach, results are typically displayed as a color overlay on a T1-weighted image of the template, which will have neither identical anatomic proportions nor identical plane of section as the subject’s untransformed brain images. Absent an additional transformation to map the quantitative results onto the subject’s native anatomy, which would carry over the inaccuracies of the aBR approach, the interpreting radiologist must interpolate the quantitative results to compensate for these differences. The sBR results, however, are displayed on the subject’s brain volume, without any spatial transformation to a template. Abnormal clusters can therefore be displayed directly on the subject’s MR images. This advantage could enhance the accuracy and efficiency of interpretation and improve correlation with clinical findings as well improving accuracy of localization for surgical planning. Because quantitative results are defined on the subject’s images, targets of interest for Magnetic Resonance Spectroscopy (MRS) or tissue biopsy based on conventional imaging could be further refined by directly targeting diffusion abnormalities. Moreover, sBR need not be limited to diffusion tensor MRI. Any quantitative or semi-quantitative imaging technique, including newer MRI diffusion techniques, dynamic susceptibility contrast MRI, dynamic contrast enhanced MRI, arterial spin labeling or MRS imaging, could leverage the advantages of sBR. In addition, sBR could improve identification of specific WM tracts affected by areas of abnormal anisotropy. Regions of abnormal FA, for example, could serve as seed ROI for tractography, thereby delineating tracts affected by disease (Fig 6).

### Limitations

A potential limitation of sBR is that it is much more computationally intensive. Using the aBR approach, a set of controls only needs to be registered once to a brain atlas template. Assuming a set of controls have previously been registered to a brain atlas template, only each new subject/patient to be analyzed need be registered to the atlas after which a quantitative voxelwise analysis can be performed by comparing to the previously processed control brain volumes. In sBR, however, where the template is the subject, all control brains must be registered to each new subject. Although considerably more registrations are thus required for sBR, parallel and high performance computing systems can reduce actual processing to be time comparable to the aBR approach.

A potential limitation of our comparison of the aBR and sBR approaches is the effect of smoothing of the imaging data as the result of transformations performed in the study. Three transformations are applied, as separate steps, using the aBR method; (1) EPI distortion correction of DTI images (within subject); (2) rigid body transformation from DTI space to T1 space (within subject) and (3) non-linear registration of the subject T1-weighted volume to the T1-weighted template. When using the sBR approach, the last transformation to the T1-weighted template is not performed for the subject of interest, but is performed for each control, to match each control’s T1-weighted volume to the subject’s. Thus, smoothing as a result of distortion correction and rigid-body transformation occur similarly in both aBR and sBR approaches; in the sBR approach one less transformation, that required for registration to the template, does not occur for the subject of interest. We performed additional analysis to characterize the ultimate impact of the single additional interpolation step on ultimate results of the analysis. The results support the conclusion that the interpolation difference does not explain the impact of the sBR approach (see S2 Appendix).

An additional limitation is that some of the available brain atlas templates are segmented atlases that can facilitate analyses. A number of software packages, which perform parcellation of an individual subject brain (e.g. Freesurfer), or registration to a segmented atlas can address this limitation.

## Conclusion

Detection of image abnormalities in a single subject requires comparison of that subject’s images to those of a control group. To ensure analogous brain regions are compared, subject and control images are registered to a template. Typically, a canonical atlas or a subject selected from the control group serves as the template. This type of atlas based registration (aBR) approach suffers from potentially important errors of accuracy due to misregistration of the subject’s brain to the atlas. The sBR approach results in a significant reduction of erroneous findings that arise from these registration errors. The resulting *SUbject-REgistered Quantification (SURE-Quant) analysis* can greatly facilitate utilization of quantitative image analysis in the clinic and can be applied to many quantitative imaging measures, even beyond diffusion MRI.

## Supporting Information

S1 Appendix Clustering Conditions, Type-I Error Rate and Base Rate of Abnormalities. (DOCX)

S2 Appendix Impact of Interpolation on Results of Voxelwise Analyses. (DOCX)

S1 Fig Receiver operating characteristic curves for 50, 100, 150 and 200 voxels cluster size thresholds in the aBR-JHU analysis. The corresponding areas under the curves are 0.594, 0.601, 0.578 and 0.533 demonstrating that a 100 voxel threshold yields the best differentiation between mTBI and controls.
(DOCX)

S1 Table Type-I Error Rate As Function Of Cluster Size Threshold For Two Different Voxelwise Single-Tail Significance Conditions. (DOCX)
