# Supplementary material for: Subject Based Registration for Individualized Analysis of Diffusion Tensor MRI
Source: PLoS One. 2015 Nov 18;10(11):e0142288. doi: 10.1371/journal.pone.0142288 (PMC4651497; doi:10.1371/journal.pone.0142288)
Supplement: S1 Appendix — (DOCX) [file pone.0142288.s001.docx]

**S1 Appendix: Clustering Conditions, Type-I Error Rate and Base Rate of Abnormalities**

In order to establish the expected Type-I error rate and base rates of abnormalities under our clustering conditions (single tail voxelwise significance of p=0.005 and cluster size threshold of 100 voxels), we first followed the procedure outlined in Forman et. al. [25] for addressing the problem of multiple comparisons. Forman et. al. demonstrated that when testing the null hypothesis that fMRI activation is absent statistical power to detect neuronal activity is enhanced when spatial clustering is used. Similarly, individual voxels in our subject's FA map, which deviate significantly from normal, are unlikely to form contiguous clusters by chance alone; the probability of cluster formation is higher in the presence of a disease, which affects several neighboring pixels.

Forman et. al. determined probabilities for chance cluster formation using 2D slices, rather than 3D volumes. Because the number of neighboring pixels is higher in 3D than in 2D datasets, we recomputed cluster size thresholds for our 3D analysis. We drew numbers from a normal Gaussian distribution and filled a 128x128x75 matrix corresponding to our 2mm isotropic resolution DTI acquisition. We then interpolated this matrix to 1mm isotropic resolution and applied a brain mask to emulate the analysis over a T1W template. Voxels exceeding 2.575 standard deviations (single tail, p-value of 0.005) were allowed to form clusters. This Monte Carlo simulation was repeated 25,000 times to obtain chance cluster rates. The resultant probabilities to observe at least one cluster larger than a specific cluster size threshold are given in S1 Table. For completeness, the table also includes type-I error rates for a voxelwise single tail p-value threshold of 0.01 (2.325 standard deviations).

This simulation demonstrates that the 100 voxel cluster size threshold we have employed is conservative and leads to a Type-I error rate for cluster formation well below 1%. However, several additional factors must be considered. First, as noted by Forman et al., the presence of spatial correlation in the DTI data, such as that introduced by the acquisition sequence or image reconstruction procedures may elevate the Type I error rate. Despite the rectangular K-space sampling employed in our DTI acquisition, SENSE reconstruction may introduce such spatial correlation. Thus, a larger cluster size threshold than what would be expected based on independent measurements [25] may be required because the shape of the relevant correlation function is difficult to estimate and incorporate into the simulation [25] in order to obtain more realistic thresholds. Additionally, noise in the FA measurement might not be Gaussian; in this case calculations may be inappropriate, especially because we are dealing with tails of the distributions, which are very sensitive to shape.

Perhaps the most important basis for considering a more conservative cluster size threshold is the inherent imperfection of spatial co-registration on which the comparison of images depends. To demonstrate this phenomenon, we selected two independent groups of 20 healthy subjects each. The first set of 20 subjects we labeled the “reference group” while the second set of 20 subjects we labeled the “test group”. When comparing each subject from the test group to the reference group, we expected to detect low FA clusters in few subjects. Specifically, because the Type-I error is below 1%, we expected to reject the null hypothesis, with probability 98%, in at most one of the 20 test subjects. In other words, seeing 2 or more test subjects fail the null hypothesis should be highly unlikely. In fact, low FA clusters were detected in 18 subjects when employing the aBR approach and in 10 subjects with the sBR approach. Thus, although the sBR approach reduced the number of false inferences, the observed rate of abnormalities appears higher than expected. As a result, the cluster size threshold optimal for the question of group discrimination must be operationally defined.

We therefore performed an additional analysis to determine the specific cluster size threshold optimal for group discrimination. One previously adopted parameter, also very commonly applied to the assessment of diagnostic tools in general, is the area under the receiver operating characteristic (ROC) curve [15]. We therefore plotted ROC curves, based on the lesion volume distributions for the test and mTBI groups, obtained at cluster size thresholds of 50, 100, 150 and 200 (S1 Fig.) and confirmed that area under the curve (AUC) is largest when the 100 voxel cluster size threshold is applied, with a 50 voxel threshold next most effective.

It remains possible that the optimal cluster size threshold varies based on the template (e.g., between different atlas templates). Most importantly, however, we applied the same threshold value (100 voxels) throughout all analysis, leaving the choice of the template as the only source of variance in our results.
