# Supplementary material for: Subject Based Registration for Individualized Analysis of Diffusion Tensor MRI
Source: PLoS One. 2015 Nov 18;10(11):e0142288. doi: 10.1371/journal.pone.0142288 (PMC4651497; doi:10.1371/journal.pone.0142288)
Supplement: S2 Appendix — (DOCX) [file pone.0142288.s002.docx]

**S2 Appendix: Impact of Interpolation on Results of Voxelwise Analyses**

We have demonstrated that imperfect registration of the subject’s data to a template leads to a bias, which is dramatically reduced by using subject’s T1-weighted image as the template. In doing so, we must address a technical detail associated with this change: the subject’s data does not undergo interpolation intrinsic to the nonlinear registration operation whereas controls’ data does. In addition, because interpolation is known to act as a filter, slightly smoothing the data, we must demonstrate that absence of the interpolation cannot explain the observed reduction of detected low FA regions, leaving the choice of template as the only plausible cause. Therefore, we introduced an artificial registration step causing interpolation of the subject’s data and repeated the rest of the cluster analysis. We displaced the subject’s T1-weighted image by half a voxel in all directions and then employed it as a template for the sBR analysis. The data from all mTBI and test subjects were analyzed in this manner, each in comparison to the test group, and results were compared to the original sBR analysis, which entails only two interpolation steps for the subject of interest.

Using the number of abnormally low FA clusters within white matter as the comparison metric, in 12 of the mTBI subjects cluster count did not change, in 2 mTBI subjects number of clusters increased by 1, in 4 mTBI subjects it decreased by 1 and in 2 mTBI subjects it decreased by 2. Pair-wise Wilcoxon signed-rank test comparing cluster counts with and without this third interpolation step does not reveal a significant difference (W=22, N=8, p=0.30). Similarly, in 14 of the test subjects, the cluster count did not change, in 2 test subjects the cluster count increased by 1, in 3 test subjects it decreased by 1 and in 1 test subject it increased by 1. Pair-wise Wilcoxon signed-rank test comparing cluster counts with and without this third interpolation step does not reveal a significant difference (W=3, N=6, p=0.95).

We next assessed the impact of the additional interpolation step on the determination of total volume of low FA clusters (i.e., number of abnormally low FA voxels within white matter). In mTBI subjects we found a mean decrease of 1.8 voxels (standard deviation 93 voxel) classified as abnormal with the additional interpolation step. In the test subjects, average change was an increase of 22.6 voxels (standard deviation 169 voxels). Pair-wise Wilcoxon signed-rank tests comparing the volume of abnormally low FA clusters with and without this third interpolation step does not reveal a significant difference (W=4, N=12, p=0.995 for mTBI and W=5, N=10, p=0.990 for test subjects).

Finally, we investigated whether the clusters identified, in the same subject with and without the additional interpolation step, were spatially concordant (i.e., the same anatomical location). To do so, we would need to shift clusters from the artificially displaced subject T1-weighted volume back to their original T1-weighted volume. Because this shift involves interpolation of the clusters themselves and the displacement is only half the voxel dimension, we instead assessed the intersection of clusters in the presence of the shift induced by the artificial registration step. This approach will underestimate the spatial concordance volume fraction and therefore the Dice index. In 8 mTBI subjects and 9 test subjects, we identified abnormally low FA clusters in analyses with and without the artificial interpolation step. The average Dice index between these pairs is 0.66 and 0.54 for mTBI and test subjects respectively. There were 8 mTBI subjects and 10 test subjects where neither of the analyses identified abnormal clusters and therefore are not accounted by the Dice index although they do indicate the 2 approaches reach similar conclusions.

Therefore, considering the results from cluster counts, total lesion volumes and the Dice index, we believe that the absence of interpolation of the T1-weighted volume of the subject has minimal effect on the results and cannot account the dramatic drop in number of clusters and their total volume when changing from aBR to sBR approaches.
