# Supplementary material for: Subject Based Registration for Individualized Analysis of Diffusion Tensor MRI
Source: PLoS One. 2015 Nov 18;10(11):e0142288. doi: 10.1371/journal.pone.0142288 (PMC4651497; doi:10.1371/journal.pone.0142288)
Supplement: S1 Fig — The corresponding areas under the curves are 0.594, 0.601, 0.578 and 0.533 demonstrating that a 100 voxel threshold yields the best differentiation between mTBI and controls. (DOCX) [file pone.0142288.s003.docx]

S1 Fig. Receiver operating characteristic curves for 50, 100, 150 and 200 voxels cluster size thresholds in the aBR-JHU analysis. The corresponding areas under the curves are 0.594, 0.601, 0.578 and 0.533 demonstrating that a 100 voxel threshold yields the best differentiation between mTBI and controls.
