# Supplementary material for: Subject Based Registration for Individualized Analysis of Diffusion Tensor MRI
Source: PLoS One. 2015 Nov 18;10(11):e0142288. doi: 10.1371/journal.pone.0142288 (PMC4651497; doi:10.1371/journal.pone.0142288)
Supplement: S1 Table — (DOCX) [file pone.0142288.s004.docx]

S1 Table. Type-I Error Rate As Function Of Cluster Size Threshold For Two Different Voxelwise Single-Tail Significance Conditions

| Cluster Size Threshold | Type-I Error Rate | |
| --- | --- | --- |
|  | Voxelwise p=0.005 | Voxelwise p=0.01 |
| 1 | 1.00000 | 1.00000 |
| 19 | 1.00000 | 1.00000 |
| 20 | 0.99988 | 1.00000 |
| 25 | 0.94496 | 1.00000 |
| 29 | 0.69592 | 1.00000 |
| 30 | 0.61276 | 0.99992 |
| 31 | 0.53216 | 0.99980 |
| 32 | 0.45844 | 0.99944 |
| 33 | 0.39424 | 0.99864 |
| 35 | 0.27816 | 0.99124 |
| 40 | 0.10944 | 0.89340 |
| 43 | 0.05900 | 0.76520 |
| 44 | 0.04852 | 0.71564 |
| 45 | 0.03960 | 0.66236 |
| 48 | 0.02132 | 0.50564 |
| 49 | 0.01812 | 0.45764 |
| 51 | 0.01232 | 0.36740 |
| 52 | 0.01032 | 0.32720 |
| 53 | 0.00820 | 0.29132 |
| 55 | 0.00540 | 0.22996 |
| 56 | 0.00460 | 0.20296 |
| 60 | 0.00240 | 0.12164 |
| 61 | 0.00212 | 0.10712 |
| 62 | 0.00164 | 0.09468 |
| 66 | 0.00060 | 0.05548 |
| 73 | 0.00012 | 0.02024 |
| 78 | 0.00004 | 0.01084 |
| 79 | 0 | 0.00968 |
| 84 |  | 0.00508 |
| 100 |  | 0.00044 |
| 125 |  | 0.00004 |
| 126 |  | 0 |
